# Hypodense regions in the peripapillary region increased the risk of macular retinoschisis detected by optical coherence tomography

**DOI:** 10.3389/fmed.2022.1018580

**Published:** 2022-12-02

**Authors:** Xiangjun She, Congying Zhou, Zhi Liang, Jin Xie, Shixin Zhao, Jiwei Tao, Yun Zhang, Jianbo Mao, Yiqi Chen, Lijun Shen

**Affiliations:** ^1^The Affiliated Eye Hospital of Wenzhou Medical University, Hangzhou, China; ^2^Department of Ophthalmology, Zhejiang Provincial People's Hospital, Hangzhou, China

**Keywords:** macular retinoschisis, pathological myopia, peripapillary region, swept source optical coherence tomography, vitreoretinal surfaces

## Abstract

**Purpose:**

The purpose of the present study was to investigate the clinical features of peripapillary regions in patients with myopic macular retinoschisis (MRS) and its association with the development of retinoschisis (RS).

**Methods:**

In this cross-sectional study, high-myopic patients with or without MRS were recruited, and the hypodense regions were analyzed in the peripapillary regions. The vitreoretinal adhesions around both macular and paravascular arcades were compared between groups. The risk factors for the development of MRS were analyzed by logistic regression.

**Results:**

Of 88 myopic eyes, MRS was detected in 45 eyes (51%). The eyes with MRS showed a higher rate of peripapillary and paravascular retinoschisis (*P* < 0.001 and *P* = 0.006). Hypodense regions were detected in 25 eyes (20.35%). Higher rates of horizontal and vertical macular MRS were detected in the hypodense group (*P* = 0.012 and *P* = 0.002). Lower refractive error, longer axial length, and higher rates of outer retinoschisis both in horizontal and vertical macular regions were observed in the hypodense group (*P* = 0.012, *P* = 0.006, *P* = 0.038, and *P* = 0.034). Higher rates of inner and outer retinoschisis, vitreoschisis, and microfolds along superior vascular arcade were detected in the hypodense group (*P* = 0.005, *P* = 0.001, *P* = 0.014, and *P* = 0.014). Higher rates of internal limiting membrane (ILM) detachment, inner and outer RS were detected along the inferior vascular arcade in the hypodense group (*P* = 0.008, *P* = 0.001, and *P* = 0.028). Hypodense regions, the axial length and PICC (peripapillary intrachoroidal cavitation) were significantly correlated with the severity of MRS (Odds ratio = 0.207, *P* = 0.010; Odds ratio = 1.399, *P* = 0.016; Odds ratio = 0.142, *P* = 0.010).

**Conclusions:**

The hypodense regions were likely to affect outer retinoschisis both in macular and paravascular regions. It was a risk factor for the development of MRS.

## Introduction

Myopic macular retinoschisis (MRS) is one of the most common diseases that endangers vision acuity in patients with pathological myopia, with its prevalence ranging from 14.7 to 34.4% (1–3). MRS is featured by the splitting of the retina, mainly located in the outer plexiform layer. The natural progress of MRS depends heavily on the severity of the disease, nearly 42.9% eyes with MRS falling under a higher grade would deteriorate in a short time (4). Nearly 2.9–31% eyes with higher MRS may progress to macular hole and 3.4–37.5% eyes may come to the final stage of retinal detachment (3–8).

Based on the previous studies, the main pathogenesis of MRS is attributed to the abnormal tractional forces to the retina from the tangential and anteroposterior orientations. Previous studies reported that posterior staphyloma (PS) was detected in 86.0% eyes with MRS, and the retinoschisis of the outer plexiform layer was all located within the PS (9). Vanderbeek and associates pointed out that perifoveal posterior vitreous detachment (PVD) was the main reason for the MRS (10). During the PVD, the vitreous cortex remnants retained above the macular surface in 40.5% high-myopic eyes (11). Kyoto Ohno-Matsui found that paravascular vitreal adhesion was another risk factor for the MRS (12). Abnormal PVD occurred earlier along the vascular arcades and the forces were transmitted to macular arcade and consequently formed the MRS (12).

The main therapy for MRS is to relieve tractional forces by vitrectomy combining with peeling the internal limiting membrane (ILM) (10). However, the main surgical complication postsurgery was the macular hole retinal detachment (13), which was nearly 36% and 40% intraoperatively and postoperatively, respectively (14–16). Thus, the exact mechanism of MRS is still unclear and the success rate of surgery needs to be largely improved, especially in the case of serious MRS.

Recently, Koh-Hei Sonda reported that the eyes with MRS were completely recovered by peeling the ILM around the peripapillary (14) and suggested a novel mechanism of abnormal adhesion from the peripapillary regions in the development of MRS. In our study, we found that the hypodense region in patients with MRS was closely related to the degree of MRS. Our study presented the features of a hypodense region in MRS and analyzed its association with abnormal vitreoretinal adhesions, both in the macular and vascular arcades for the purpose of ascertaining its in-depth role in the pathology of MRS.

## Materials and methods

### Subjects

The present study was approved by the Institutional Research Committee of the Affiliated Eye Hospital of the Wenzhou Medical University (Zhejiang, China). It was carried out according to the guidelines mentioned in the World Medical Association's Declaration of Helsinki. Each participant was informed of the content and purpose of the study before giving consent to take part in the study. Our study included those patients with high myopia who underwent treatment for the same defect at the Vitreoretinal Center of Eye Hospital Affiliated to the Wenzhou Medical University from 2019 to 2021. The inclusion criteria were as follows: (1) high-myopic eyes, the spherical equivalent refractive error of the eye should have been <-6.0 Diopters and the axial length >26.5 mm; (2) should have been ready to undergo complete eye examinations, including best corrected visual acuity (BCVA), intraocular pressure (IOP), refractive error, axial length (AL) measurement, fundus photographs, and Swept Source Optical Coherence Tomography (SS-OCT) imaging. The eye of the normal volunteers without high myopia was selected as the control group. The inclusion criteria were as follows: (1) the spherical equivalent refractive error of the eye should have been >-6.0 Diopters and the axial length <26.5 mm, (2) should have been without any history of macular, glaucoma, and uveitis disease, and (3) should be ready to undergo a normal IOP measurement.

### Comprehensive ophthalmological examination

The clinical baseline data were age, gender, BCVA, and refractive error by subjective refraction. The BCVA was determined by a Standard Logarithmic Visual Acuity Chart and converted to logarithm of the minimum angle of resolution (log MAR) units for the statistical analyses. All patients accepted to undergo comprehensive ophthalmological examinations, during which the intraocular pressure was measured by a Non-Contact Tonometer (NCT, Topcon Corporation, Tokyo, Japan), the axial length was measured by the IOL Master (Carl Zeiss Meditec), Ultra-widefield Optos was captured by ULTRA-WIDEFIELD FUNDUS IMAGING (Optos 200Tx Capturing Technique, Optos PLC, Dunfermline, Scotland, UK), and a slit-lamp was used to examine the ocular conditions from anterior to posterior part. The presence of staphyloma was determined by the presence of abnormalities in pigmentation and abrupt thinning of the scleral margin around the border of a staphyloma, as mentioned in a previous study (17).

### Swept source optical coherence tomography (SS-OCT) image acquisition and analysis

The optical coherence tomography (OCT) images were obtained from the SS-OCT system (VG200D, SVision Imaging, Ltd., Luoyang, Henan, China), with the wavelength of 1,050 nm and a combination of the industry-leading specifications. The scan speed was 200,000 A scans per second, a wide field was of 56 degrees, and the axial resolution was 5 μm. Thirty-three line scans were performed in the macula region through horizontal and vertical orientations, superior and inferior vascular arcades, and peripapillary regions separately.

Well-qualified OCT images were chosen for the analysis. Two experienced retina specialists were asked to judge the images, while another senior retina specialist was asked to make the final decision on the inconsistent judgments. The inner retinoschisis was defined as a splitting between the ILM and the ganglion cell layer, the outer retinoschisis was defined as a splitting of the outer plexiform layer, while ILM detachment was defined as the detachment of the inner limiting membrane (ILM) from the inner surface of retina (6). The retinoschisis was evaluated by two skilled ophthalmologists. The MRS was defined as the retinoschisis in the macular region; otherwise, it signified the absence of MRS. The Grade of macular retinoschisis (MRS) was judged, according to the previous study by Shimada: S0 for no macular retinoschisis; S1 for extrafoveal macular retinoschisis; S2 for fovea-only macular retinoschisis; S3 for foveal but not the whole macular area with macular retinoschisis; and S4 for the entire macular area with macular retinoschisis (4). However, as there was no standard to evaluate the RS outside the macular region, if the RS was found outside the macular region, it belonged to S1 in the absence of the MRS group. The incidence of ILM detachment, inner retinoschisis, and outer retinoschisis was judged at the horizontal and vertical macular regions, respectively.

Paravascular abnormalities, including microcysts, microhole, and microfolds along the superior and inferior arcades, were observed. Paravascular microcysts were observed as spindle-shaped, dark areas or fissure-like lesions adjacent to retinal vessels (2). Microfolds were a tent-like traction of retinal vessels to vitreous cavity (18). Microholes were the cracks that extended from the level of the internal limiting membrane (ILM) to approximately one half of the thickness of the neural retina and also named “paravascular lamellar hole” (2, 19).

**Figure 1:**
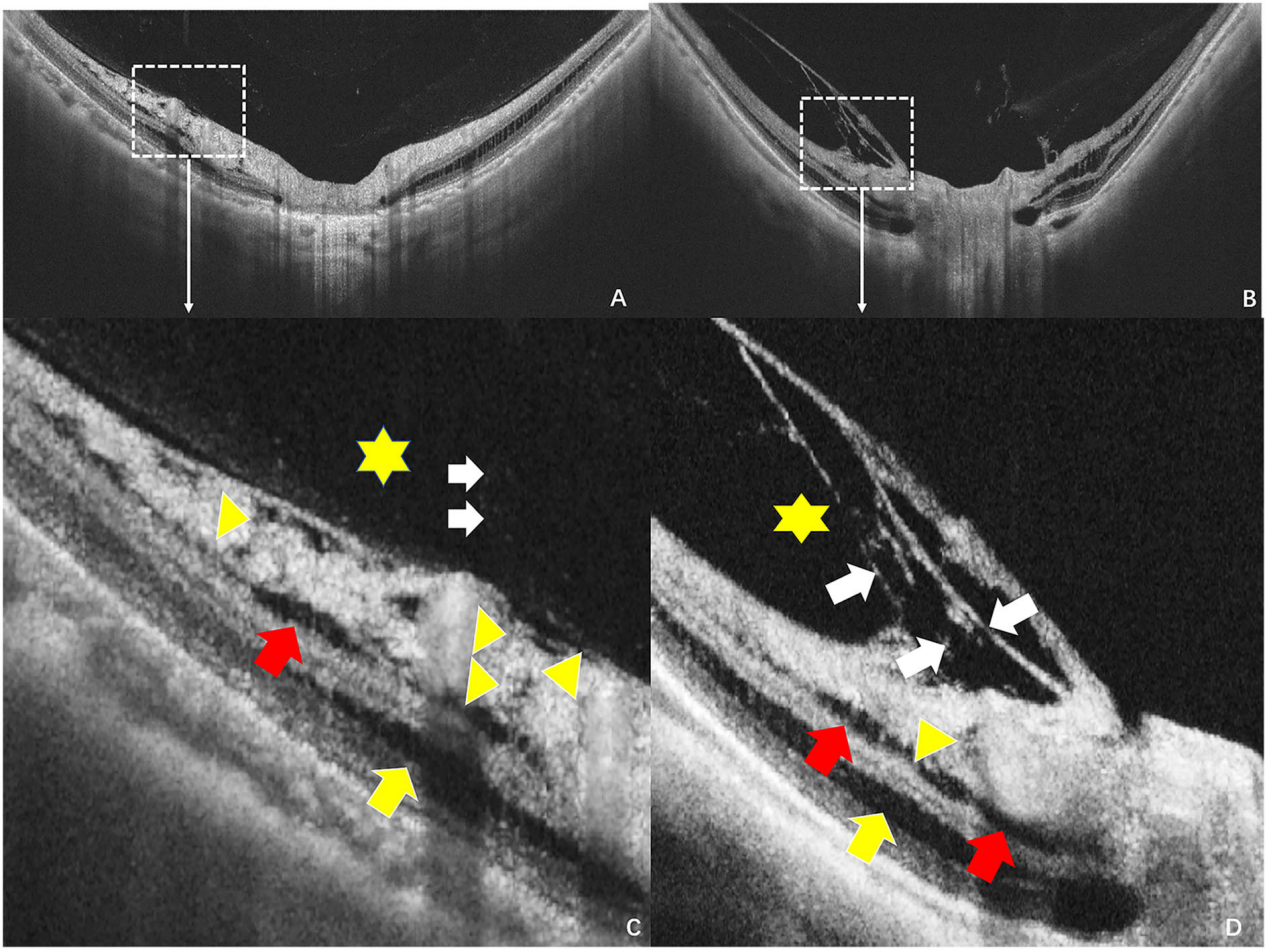
Hypodense regions and vitreoretinal interfaces in the peripapillary regions by swept source optical coherence tomography (SS-OCT). **(A,B)** Images obtained by scanning the peripapillary regions at the vertical orientation, **(C,D)** separately stands for the enlarged images of the white box from the A,B. **(C)** The yellow triangle indicates the hypodense region across the retina, a low reflection crevice structure was detected between the arrowhead that crossed the retina from the defective vitreous cortex to the inner retina, the red arrow stands for the inner retinoschisis, yellow arrow stands for the outer retinoschisis, the asterisk stands for the liquefication cavity next to the hypodense regions, and white arrow stands for the posterior vitreous. **(D)** The yellow triangle indicates the hypodense region, red and yellow arrows separately indicate the inner and outer retinoschisis, while the white arrow indicates vitreoschisis.

Hypodense regions were small round regions or fissure (hypodense) reflectance in inner retina, especially in the retinal nerve fiber layer (RNFL). A low reflection crevice structure was detected in the superficial RNFL with a defective vitreous cortex near the retina, according to the previous study (20) (Figure 1). The integrity of ILM and vitreous liquefication were evaluated near the hypodense regions (Figure 1).

Peripapillary intrachoroidal cavitation (PICC) was defined as the localized orange peripapillary elevation of the peripapillary retinal pigment epithelium (RPE) (21), which was identified as the peripapillary detachment in pathologic myopia (PM) (22) or subarachnoid space around the optic nerve (23).

### Statistical analysis

The incidence of MRS, including the ILM detachment, inner and outer retinoschisis around the peripapillary regions were compared in eyes with or without hypodense regions in peripapillary. Age, BCVA, axial length, refractive error, and paravascular vitreal adhesion were compared between the eyes with and without hypodense regions using an independent *t*-test. The incidence was compared using chi-square tests or Fisher's exact probability tests between two groups. Logistic regression was used to analyze the risk factors related to MRS. The presence of hypodense regions, age, AL, and PICC were treated as the independent variables, and the Grade of MRS (eyes with S0 as 0, S1 as 1, S2 as 2, S3 as 3, and S4 as 4) as the dependent variable. A *P*-value of <0.05 was considered as significant. All the data were analyzed by SPSS software version 25.0 (SPSS Inc., Chicago, IL, USA).

## Results

A total of 127 eyes (92 patients) with high myopia and 35 eyes (30 normal persons) were enrolled in the study. Four eyes (3.2%) were excluded from the study on account of poor OCT images (0.8%), rhegmatogenous retinal detachment (0.8%), macular holes (0.8%), and choroidal neovascularization (0.8%). Eighty-eight eyes with high myopia and 35 normal eyes were finally included in our study. The accuracy of judgment for MRS was 96%. A senior ophthalmologist was asked to make the final decision on the disputed images. The mean age is 54.3 ± 12.4 years old (ranging from 26 to 84 years), the mean AL is 27.89 ± 3.27 mm (ranged from 21.67 to 34.36 mm), and the mean refractive error is −10.94 ± 7.74 D (ranged from −32.0 D to 3.75 D).

### Demographic data of patients with macular retinoschisis

Among the high-myopic eyes, 45 eyes (51%) had macular retinoschisis and 43 eyes (49%) showed the absence of MRS (Table 1). There was no significant difference in mean age (55.5 ± 11.5 vs. 51.6 ± 9.1 years, *P* = 0.127) and intraocular pressure (IOP) (15.98 ± 3.27 vs. 16.04 ± 3.42 mmHg, *P* = 0.931) between MRS and without the MRS group. The MRS group had worse BCVA (0.64 ± 0.51 vs. 0.32 ± 0.33, *P* < 0.001) and a lower refractive error (−15.85 ± 5.14 vs. −13.96 ± 4.78 D, *P* = 0.047) when compared with the group without MRS. The mean axial length of the MRS group was similar to that without MRS (29.67 ± 1.62 vs. 29.58 ± 2.20 mm, *P* = 0.818), but it was much longer than for normal people (*P* < 0.001). Posterior staphyloma was detected in most of the eyes with or without MRS group (77.8 vs. 81.4%, *P* = 0.674). Macular, vascular arcades, and peripapillary lesions were compared between the MRS group and without MRS group in Table 1. We found that the retinoschisis was detected across the entire macula (Grade S4) in 27 eyes (60.0%) with MRS, followed by the 10 eyes (22.2%) of extra-foveal macular retinoschisis (Grade S1), 7 eyes (15.6%) of foveal but not the entire macular area macular retinoschisis (Grade S3), and 1 eye (2.2%) of fovea-only macular retinoschisis (Grade S2). More superior and inferior paravascular and peripapillary retinoschisis were detected in the eyes with than without the MRS group (Superior: 66.6 vs. 4.7%, *P* < 0.001; inferior: 66.6 vs. 6.7%, *P* < 0.001; Peripapillary: 28.9 vs. 9.3%, *P* = 0.006).

**Table 1:** Characteristics of patients with and without myopic macular retinoschisis.

	**MRS**		***P*** **-value**	**Normal**	***P-*** **value**
	**Absent**	**Present**			
**No. of eyes**	43	45		35	
Male	13	10		6	*0.385*
Female	30	35		29	
**Age (year)**					
Mean ± SD	51.6 ± 9.1	55.5 ± 11.5	*0.127*	56.7 ± 15.3	*0.120^a^*
Range	39–75	31–79		*26–84*	
**Intraocular pressure (mmHg)**					
Mean ± SD	16.04 ± 3.42	15.98 ± 3.27	*0.931*	14.27 ± 2.67	*0.014*
Range	10.0–26.0	11.3–26.5		9.6–19.6	
**BCVA**					
Mean ± SD	0.32 ± 0.33	0.64 ± 0.51	* < 0.001*	0.12 ± 0.18	* < 0.001^a^*
Range	0–1.30	0–2.30		0–0.82	
**Refractive error (D)**					
Mean ± SD	−13.96 ± 4.78	−15.85 ± 5.14	*0.047*	−0.93 ± 2.48	* <0.001^a^*
Range	−22.50 to −4.88	−32.00 to −6.50		−6.63 to 3.75	
**Axial length (mm)**					
Mean ± SD	29.58 ± 2.20	29.67 ± 1.62	*0.818*	23.54 ± 1.36	* <0.001^a^*
Range	26.03–34.36	25.27–34.13		21.67–25.85	
**MRS grade**					
S0	35 (81.4%)	0 (0.0%)		35 (100.0%)	
S1	8 (18.6%)	10 (22.2%)		0 (0.0%)	
S2	0 (0.0%)	1 (2.2%)		0 (0.0%)	*<0.001*
S3	0 (0.0%)	7 (15.6%)		0 (0.0%)	
S4	0 (0.0%)	27 (60.0%)		0 (0.0%)	
**Posterior staphyloma**					
Present	35 (81.4%)	35 (77.8%)	*0.674*	0 (0.0%)	*<0.001*
**Paravascular retinoschisis**					
Superior	2 (4.7%)	30 (66.6%)	*<0.001*	0 (0.0%)	*<0.001*
Inferior	3 (6.7%)	30 (66.6%)	*<0.001*	0 (0.0%)	*<0.001*
**Peripapillary retinoschisis**					
Present	4 (9.3%)	13 (28.9%)	*0.006*	0 (0.0%)	*<0.001*

MRS stands for macular retinoschisis, BCVA stands for best corrected vision acuity, a value of P < 0.05 was considered as significant,

a stands for an independent t-test, others are chi-square test for the analysis.

### Characteristics of patients with peripapillary hypodense regions

Hypodense regions in the peripapillary were observed in 25 eyes (20.3%) in Table 2. Higher rates of horizontal and vertical MRS were detected in the eyes with hypodense regions than those without hypodense regions (10.66 vs. 9.84%, *P* = 0.012) (12.3 vs. 8.2%, *P* = 0.002). There were no significant differences in age, intraocular pressure, and BCVA between the eyes with or the eyes without hypodense regions (*P* = 0.625; *P* = 0.510; and *P* = 0.077). Lower refractive error (−13.36 ± 4.48 D vs. −10.18 ± 8.29 vs, *P* = 0.012) and longer AL (29.50 ± 1.69 vs. 27.47 ± 3.4 mm, *P* = 0.006) were detected in the eyes with the hypodense region in Table 2. Most of the eyes without hypodense region were divided into lower Grades of MRS, such as 62 eyes (50.82%) for S0 and 13 eyes (10.66%) for S1. The degrees of MRS in the hypodense region group were S4 (9.84%), S0 (6.56%), and S1 (4.1%) (*P* = 0.002). The retinoschisis in inferior vascular arcades were detected more than that in superior (10.66% vs. 9.84%, *P* = 0.002). The eyes with peripapillary retinoschisis were slightly lower for the hypodense region group than those without hypodense regions (9.02 vs. 10.66%).

**Table 2:** Characteristics of patients with and without hypodense regions.

**No. of eyes**	**Hypodense regions**		***P*** **-value**
	**Absent**	**Present**	
Male	21 (17.2%)	8 (6.5%)	*0.273*
Female	76 (62.3%)	17 (13.9%)	
**Age (year)**			
Mean ± SD	54.44 ± 12.78	53.08 ± 10.78	*0.625^a^*
Range	26–84	31–68	
**Intraocular pressure (mmHg)**			
Mean ± SD	15.60 ± 3.60	15.08 ± 2.50	*0.510*
Range	9.6–26.5	11.3–21.0	
**BCVA**			
Mean ± SD	0.38 ± 0.43	0.36 ± 0.43	*0.077^a^*
Range	0–2.30	0–1.85	
**Refractive error (D)**			
Mean ± SD	−10.18 ± 8.29	−13.36 ± 4.48	*0.012^a^*
Range	−32.00 to −3.75	−21.63 to −6.63	
**Axial length (mm)**			
Mean ± SD	27.47 ± 3.47	29.50 ± 1.69	*0.006^a^*
Range	21.67 ± 34.13	26.03 ± 34.36	
**MRS grade**			
S0	62 (50.82%)	8 (6.56%)	*0.002*
S1	13 (10.66%)	5 (4.1%)	
S2	1 (0.82%)	0 (0.00%)	
S3	7 (5.74%)	0 (0.00%)	
S4	14 (11.48%)	12 (9.84%)	
**MRS (horizontal)**			
Present	25 (20.49%)	13 (10.66%)	*0.012*
Absent	72 (59.02%)	12 (9.84%)	
**MRS (vertical)**			
Present	71 (58.20%)	15 (12.30%)	*0.002*
Absent	26 (21.31%)	10 (8.20%)	
**Posterior staphyloma**			
Present	49 (40.16%)	21 (17.21%)	*0.003*
Absent	48 (39.34%)	4 (3.28%)	
**Paravascular retinoschisis**			
Superior	19 (15.57%)	12 (9.84%)	*0.004*
Inferior	20 (16.39%)	13 (10.66%)	*0.002*
**Peripapillary retinoschisis**			
Present	13 (10.66%)	11 (9.02%)	*0.001*
Absent	84 (68.85%)	14 (11.48%)	
**PICC**			
Present	11 (9.02%)	3 (2.46%)	*0.700*
Absent	86 (70.49%)	22 (18.03%)	

MRS stands for macular retinoschisis, BCVA stands for best corrected vision acuity, PICC stands for peripapillary intrachoroidal cavitation; a value of P < 0.05 was considered as significant,

a stands for an independent t-test, while others are chi-square test for the analysis.

### Higher rates of retinoschisis at macular and vascular arcades with hypodense regions

Among the high-myopic eyes, horizontal MRS, vertical MRS, and paravascular retinoshisis were separately detected in 39 eyes (31.7%), 42 eyes (34.1%), and 49 eyes (39.8%). The types of retinoschisis were compared between the eyes with or those without hypodense regions in Table 3 and Supplementary Figure S1. A higher rate of outer retinoschisis was detected in the eyes with hypodense regions than those without hypodense regions, either in horizontal or vertical macular. (Horizontal: 10.2 vs. 4.5%, *P* = 0.038, vertical: 11.4 vs. 3.4%, *P* = 0.034); however, there were no significant differences in the ILM detachment and inner RS and IRS + ORS + ILM detachment at the horizontal macular (ILM: 10.2 vs. 4.5%, *P* = 0.073; inner retinoschisis: 12.5 vs. 2.3%, *P* = 0.638; IRS + ORS + ILM detachment: 13.6 vs. 1.1%, *P* = 0.275) in Table 3. Higher rates of ILM detachment and ILM + IRS + ORS were detected in the group with hypodense regions than the group without hypodense regions (8.0 vs. 6.8%, *P* = 0.001, 10.2 vs. 4.5%, *P* = 0.017).

**Table 3:** Types of retinoschisis of eyes with and without hypodense regions.

	**Hypodense regions**		***P*** **-value**
	**Absent**	**Present**	
**Horizontal macular**			
ILM detachment	9 (10.2%)	4 (4.5%)	*0.073*
IRS	11 (12.5%)	2 (2.3%)	*0.638*
ORS	4 (4.5%)	9 (10.2%)	*0.038*
ILM + IRS + ORS	12 (13.6%)	1 (1.1%)	*0.275*
**Vertical macular**			
ILM detachment	7 (8.0%)	6 (6.8%)	*0.001*
IRS	8 (9.1%)	5 (5.7%)	*0.326*
ORS	3 (3.4%)	10 (11.4%)	*0.034*
ILM + IRS + ORS	9 (10.2%)	4 (4.5%)	*0.017*
**Superior vascular**			
ILM detachment	8 (9.1%)	5 (5.7%)	*0.014*
IRS	5 (5.7%)	8 (9.1%)	*0.005*
ORS	3 (3.4%)	10 (11.4%)	*0.010*
Vitreoschisis	6 (6.8%)	7 (8.0%)	*0.014*
Microfolds	1 (1.1%)	12 (13.6%)	*0.014*
**Inferior vascular**			
ILM detachment	6 (6.8%)	7 (8.0%)	*0.008*
IRS	3 (3.4%)	10 (11.4%)	*0.001*
ORS	3 (3.4%)	10 (11.4%)	*0.028*
Vitreoschisis	7 (8.0%)	6 (6.8%)	*0.098*
Microfolds	2 (2.3%)	11 (12.5%)	*0.122*

IRS means inner retinoschisis, ORS means outer retinoschisis, ILM means inner limiting membrane, a value of p < 0.05 was considered as significant using the chi-square test.

In the eyes with hypodense regions, the rates of IRS and ORS were much higher than those of the eyes without hypodense regions both in superior and inferior vascular arcades (IRS: 9.1 vs. 5.7%, *P* = 0.005, 11.4 vs. 3.4%, *P* = 0.001, ORS: 11.4 vs. 3.4%, *P* = 0.010, 11.4 vs. 3.4%, *P* = 0.028) (Table 3). However, at the superior vascular arcade, the rate of ILM detachment in the eyes with the hypodense region group was less than that in eyes without the hypodense region group (5.7 vs. 9.1%, *P* = 0.014), and it was opposite at the inferior vascular arcade (8.0 vs. 6.8%, *P* = 0.008). Furthermore, higher rates of vitreoschisis and microfolds were detected in eyes with hypodense regions than in eyes without hypodense regions (vitreoschisis: 8.0 vs. 6.8%, *P* = 0.014; microfolds: 13.6 vs. 1.1%, *P* = 0.014) along the superior vascular arcade in Table 3 and Supplementary Figure S2A. Higher rates of IRS, ORS, and IRS + ORS coexisted along vascular arcades were detected in eyes with hypodense regions.

### Development of macular retinoschisis with the presence of hypodense regions

**Figure 2:**
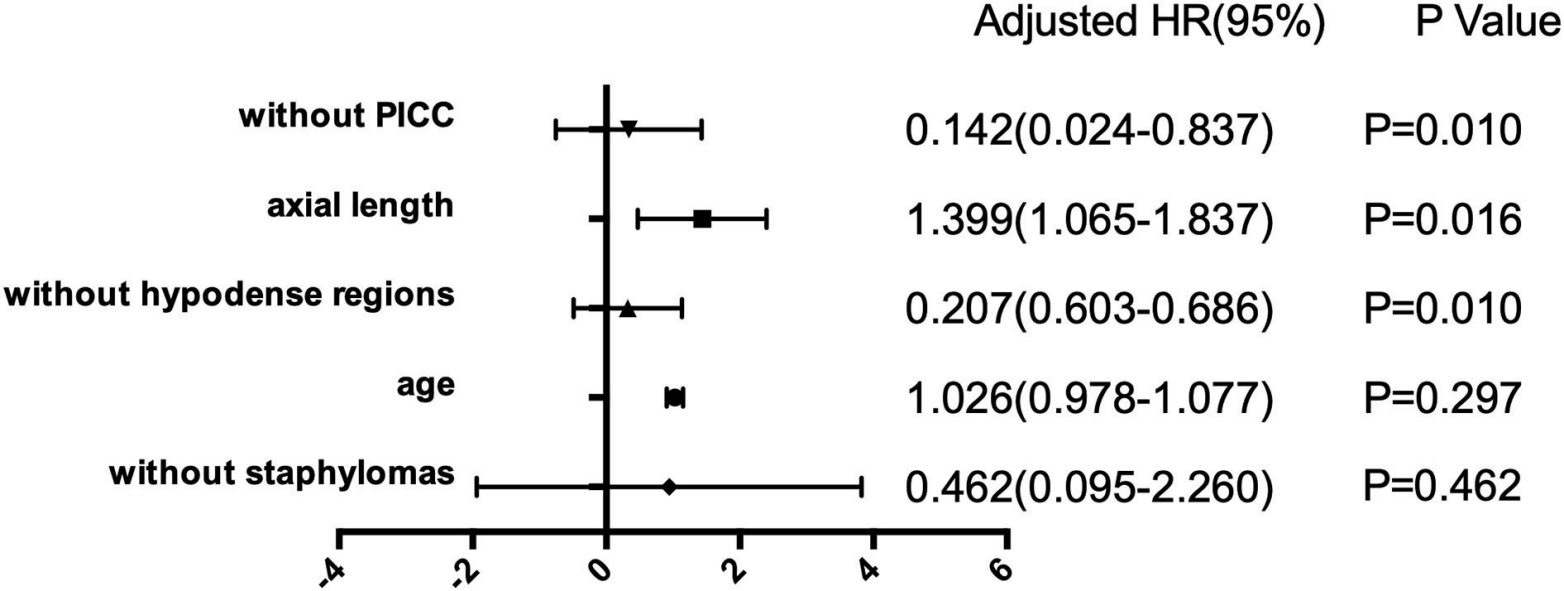
Forest plots for the risk factors with the severity of macular retinoschisis (MRS). Horizontal lines stand for odds ratios (ORs) and the corresponding 95% confidence intervals (CIs), *p*-value is given in the right box. Generalized linear model (GLM) was used to analyze the risk factors related to the development of MRS.

Logistic regression was used to evaluate the evolution of hypodense regions to the degrees of macular retinoschisis. The results revealed that hypodense regions (Odds ratio = 0.207, 95% confidence interval (CI): 0.603–0.686, *P* = 0.010), axial length (Odds ratio = 1.399, 95% confidence interval: 1.065–1.837, *P* = 0.016), and PICC (Odds ratio = 0.142, 95% confidence interval: 0.024–0.837, *P* = 0.010) were significantly correlated with degrees of macular retinoschisis, as presented in Figure 2.

## Discussion

Our study showed that peripapillary hypodense regions were a risk factor for the degree of macular retinoschisis. MRS with hypodense regions showed higher rates of outer retinoschisis both in the horizontal and vertical macular orientations. A higher prevalence of paravascular abnormalities around the vascular arcade was found in MRS eyes with hypodense regions. The present study described the distributions of vitreoretinal surfaces in patients with hypodense regions in MRS. It was the first study to present the prevalence of hypodense regions in patients with MRS, which may reveal the role of hypodense regions in the development of macular retinoschisis.

Hypodense regions look like a fissure of low or absent reflectance in the inner retina, especially in RNFL (20). They were always detected in patients with glaucoma, with nearly 16% of the glaucomatous eyes (14, 20). The prevalence is (20.3%) in patients with pathological myopia in our study, significantly higher than those of previous studies in patients with glaucoma. No hypodense regions were detected in normal people. A longer axial length and higher refractive error were reported in eyes with hypodense regions, as presented in Table 2. Brad Fortune reported that the hypodense regions were associated with high myopia and epiretinal membranes (ERM) in glaucoma patients (24). He suggested that the elongation of axial length may be a risk factor for hypodense regions.

**Figure 3:**
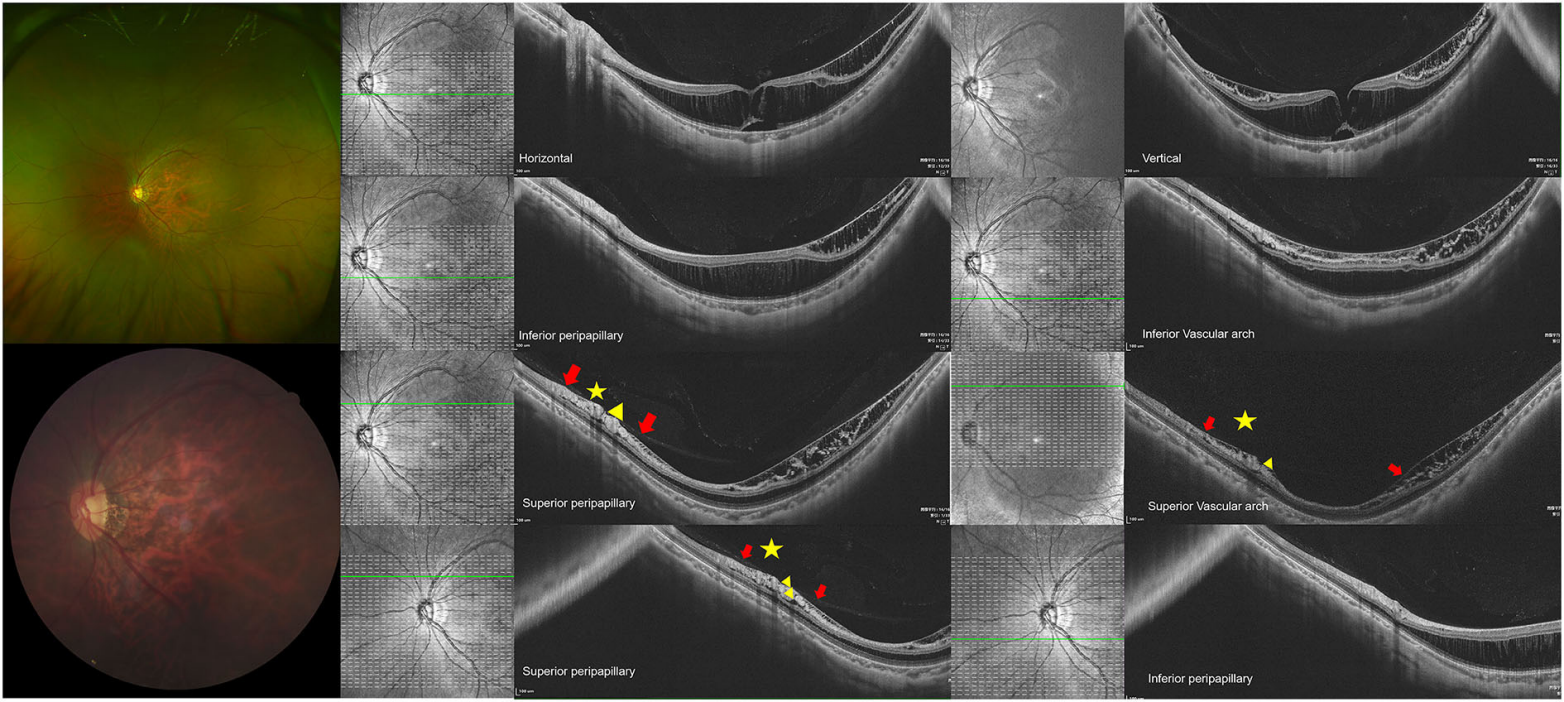
Eyes with macular retinoschisis (MRS) Showed peripapillary hypodense regions. **(Top row)** shows a 31-year-old man, with the axial length (AL) of 27.93 mm and the refractive error of minus 7 diopters. The representative images through horizontal and vertical macula are presented on the top arrow, in which the outer retinoschisis and foveal detachment were detected, internal limiting membrane (ILM) detachment and inner retinoschisis were detected at the temporal, superior, and inferior macular. **(2nd row)** Outer retinoschisis and ILM detachment were detected at the inferior peripapillary and vascular arch, while inner retinoschisis was detected at the inferior vascular arch. **(3rd row)** The inner and outer retinoschisis and ILM detachment were detected around superior peripapillary and vascular arch regions. **(Last row)** Hypodense regions were detected around superior peripapillary and vascular arch regions (yellow triangle), ILM detachment, inner and outer retinoschisis were detected around the regions. The yellow asterisk indicates the liquefication cavity, while the red arrow indicates the posterior vitreous cortex detachment.

Hypodense regions were always detected to be located adjacent to major temporal vessel branches, were more frequent in the superior peripapillary, and were associated with the paravascular RNFL defect (25). A hypothesis was proposed to show that the loss of local axons might increase the mechanical forces that pulled axons around the vessels (20). Our study found that hypodense regions were commonly detected in the superior peripapillary regions, and the prevalence of RNFL defect in the eyes with hypodense regions was 12.3% higher than in the eyes without hypodense regions. Furthermore, our study indicated that paravascular abnormal adhesion coexisted with hypodense regions along the vascular arcade, as presented in Supplementary Figure S2 and Table 3. The prevalence of microfolds and microcysts was higher in the eyes with peripapillary hypodense regions than in the eyes of those being absent, as shown in Supplementary Figure S2A. It suggested that abnormal vitreoretinal adhesion was present and may increase the lateral tension to the inner retinal surface. As Figures 1, 3 present, liquified vitreous with abnormal adhesion was adjacent to the hypodense regions and was accompanied with inner and outer retinoschisis. When liquefication occurred near the region, the integrity of ILM may have become disrupted, while another study pointed out that the vitreous fluid may migrate through the ILM and increase the lateral traction (24, 26). We supposed that when the ILM was destroyed, vitreous fluid may migrate and destroy the RNFL around the vessels, consequently forming the structure of hypodense regions.

In the present study, the rate of outer retinoschisis was higher in vascular arcades than macula in eyes with hypodense regions. The hypodense regions were frequently detected with paravascular inner retinal defects (PIRDs) along the superior vascular arcade (1, 25, 27). Shorter holes caused by the traction near vessels were considered as the earlier form of PIRD (25). Kyoko Ohno-Matsui pointed out the importance of paravascular adhesions for the development of macular retinoschisis (12). Our study found that the rates of microfolds and microcysts were higher in eyes with hypodense regions in Supplementary Figure S2. These facts suggested that abnormal vitreal adhesions may turn out to be a more serious disorder along the vascular arcades in eyes with hypodense regions. As the reason for these abnormal vitreal adhesions was not clear, the orientation of vitreous fibers was considered to be an important reason. The lamellar and perpendicularly oriented vitreous fibers were detected from the superior vascular arcade to optic regions (28). We supposed that the tight junction between vitreous fibers may bring about reams of stretch to the peripapillary regions.

**Figure 4:**
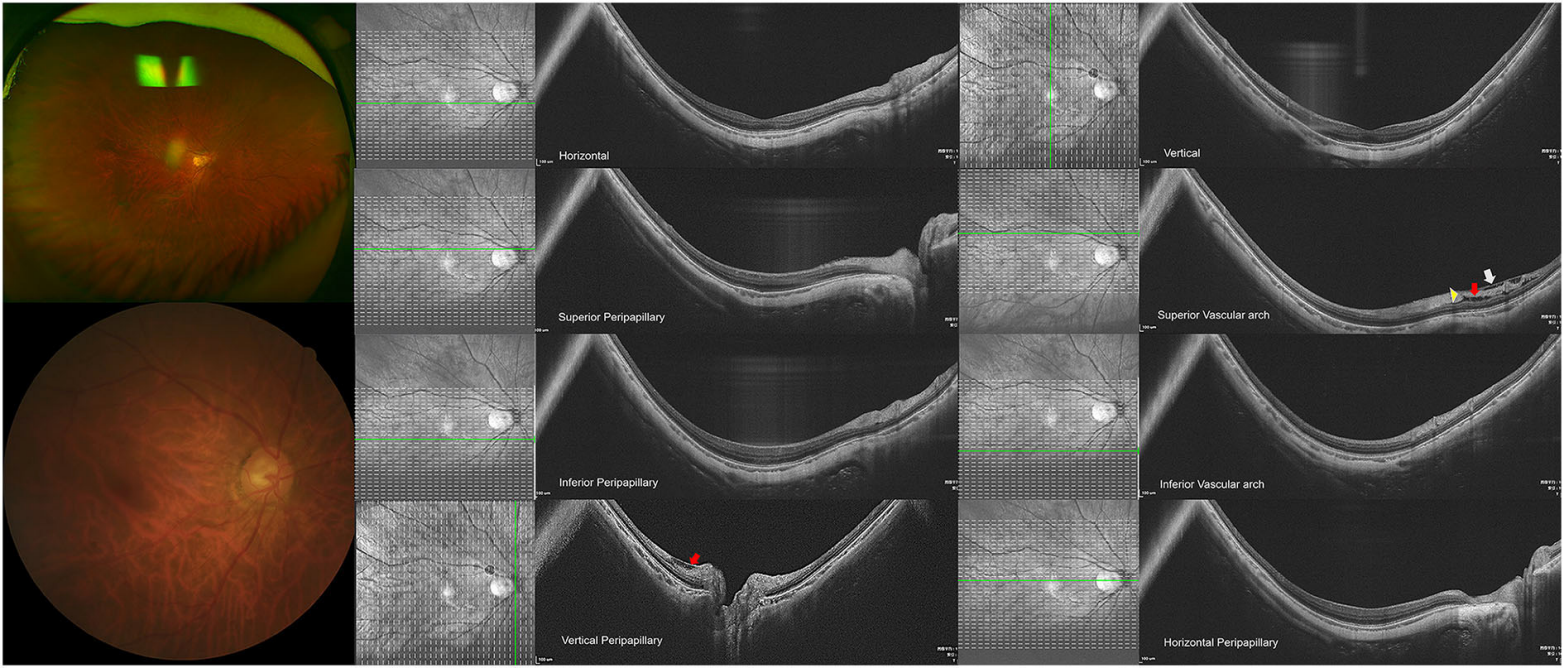
High-myopic eyes without peripapillary hypodense regions. **(Top row)** Shows a 68-year-old woman with the axial length (AL) of 31.59 mm and the refractive error of minus 19 diopters. The representative images from the horizontal and vertical macular are presented on the top arrow. **(2nd row)** Shows the images in superior peripapillary and vascular arch regions, in which the internal limiting membrane (ILM) detachment (white arrow) with inner retinoschisis (red arrow) was detected in the superior vascular arch, with hypodense region in the vascular arch (yellow triangle), **(3rd row)** indicates the images from inferior peripapillary and vascular regions. **(Last row)** Shows the images from the vertical and horizontal peripapillary regions, in which the ILM detachment and inner retinoschisis (red arrow) were detected in the superior peripapillary.

The lateral forces to the retina were considered the important reason for the development of MRS. In our study, the defect in ILM was detected, adjacent to hypodense regions in eyes with MRS as shown in Figure 4. The collagen fibrils of ILM were interwoven with the foot membrane of the Muller cells (29). ILM could be destroyed by lateral contraction from the vessels, Muller glia proliferation, and atrophy of the inner retina. Muller cells could sense and react with these forces from lateral contraction and optic nerve head (ONH) deformation, and active Muller cells may lose the ability to maintain homeostasis in the retina (30). Tractional forces increase when the integrity of ILM gets destroyed. Then, the vitreous fluid migrates, the junctions between cells decrease and finally it causes retinoschisis to develop (31). Based on the results, we explained the reason behind the successful recovery of macular retinoschisis after vitrectomy with peripapillary internal limiting membrane in the recent report (14).

**Figure 5:**
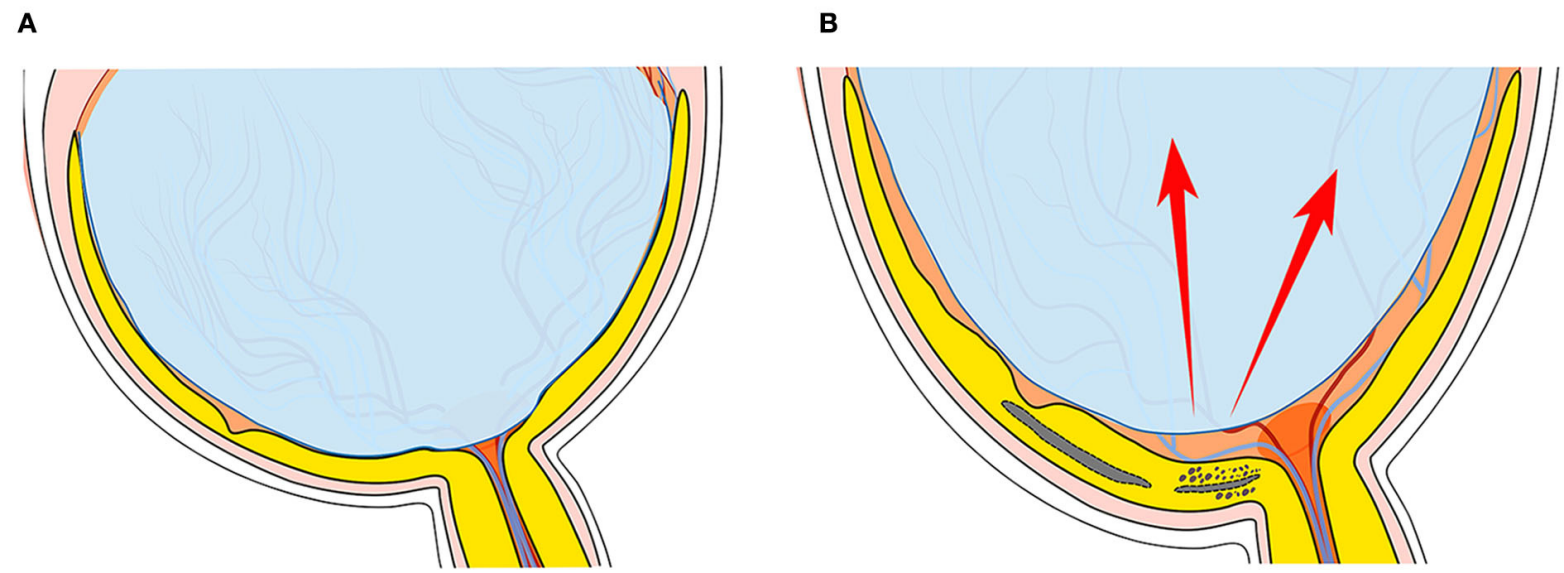
The schematic of hypodense regions in peripapillary with the development of macular retinoschisis (MRS). **(A)** Stands for a normal eye ball, while **(B)** stands for an elongated eye ball with MRS, with increasing axial length (AL), abnormal tractional forces to retinal surface increased along the AL orientation as the left red arrow points, while tangential tractional forces may be transmitted from the vascular arcade to the peripapillary region as the right red arrow indicates, which may destroy the integrity of the internal limiting membrane (ILM) in the peripapillary region as the dotted line indicates. Vitreous adhesion increased around the destroyed ILM, more forces get transmitted to the retinal surface and destroy the balance of Muller cells and consequently increased the development of MRS.

Figures 3, 4 illustrate two patients with PM, while the first patient had disrupted ILM and hypodense region in peripapillary, a higher degree of MRS was observed in the macular, the second patient had longer axial length, while the ILM was intact and without hypodense region, the macular was good. Based on these findings, we supposed that hypodense regions in peripapillary were associated with paravascular abnormal adhesions. An abnormal posterior vitreous detachment might arise from the vascular arcades and bring about much force to RNFL in peripapillary. Thus, the integrity of ILM was destroyed and the status of Mullers was altered, consequently destroying the homeostasis of the retina, vitreous fluid then migrates to the retina and accelerates the development of macular retinoschisis in Figure 5.

Our study also has limitations. First, the sample was tiny, so the MRS system in the study could not reflect the severity of the disease, especially for S0 and S1. An objective and scientific system is needed to reflect the severity of the disease in the future. Second, the results of dynamic changes in the morphology of hypodense regions associated with the development of macular retinoschisis were not available. Third, we could not evaluate the junction of ILM around the hypodense regions, like fundus fluorescein angiography (FFA) to judge the status of the hypodense regions. A further longitudinal study is necessary to reveal the in-depth role of the hypodense region in the pathology of MRS in the future.

## Data availability statement

The raw data supporting the conclusions of this article will be made available by the authors, without undue reservation.

## Ethics statement

The studies involving human participants were reviewed and approved by the Ethics Committee of the Eye Hospital Affiliated with Wenzhou Medical University. The patients/participants provided their written informed consent to participate in this study.

## Author contributions

XS and CZ conceived and designed the study. ZL and CZ collected the data and wrote the manuscript. JX and SZ collected and analyzed the data. JT, YZ, and JM provided interpretation and revision. YC and LS participated in the conception and critical revision. All authors contributed to the article and approved the submitted version.

## Funding

This project was supported by the Zhejiang Provincial Natural Science Foundation of China (LQ20H120004) and the National Natural Science Foundation of China (82101158).

## Conflict of interest

The authors declare that the research was conducted in the absence of any commercial or financial relationships that could be construed as a potential conflict of interest.

## Publisher's note

All claims expressed in this article are solely those of the authors and do not necessarily represent those of their affiliated organizations, or those of the publisher, the editors and the reviewers. Any product that may be evaluated in this article, or claim that may be made by its manufacturer, is not guaranteed or endorsed by the publisher.
