# Supplementary figures and images for: Hypodense regions in the peripapillary region increased the risk of macular retinoschisis detected by optical coherence tomography
Source: Front Med (Lausanne). 2022 Dec 2;9:1018580. doi: 10.3389/fmed.2022.1018580 (PMC9755345; doi:10.3389/fmed.2022.1018580)

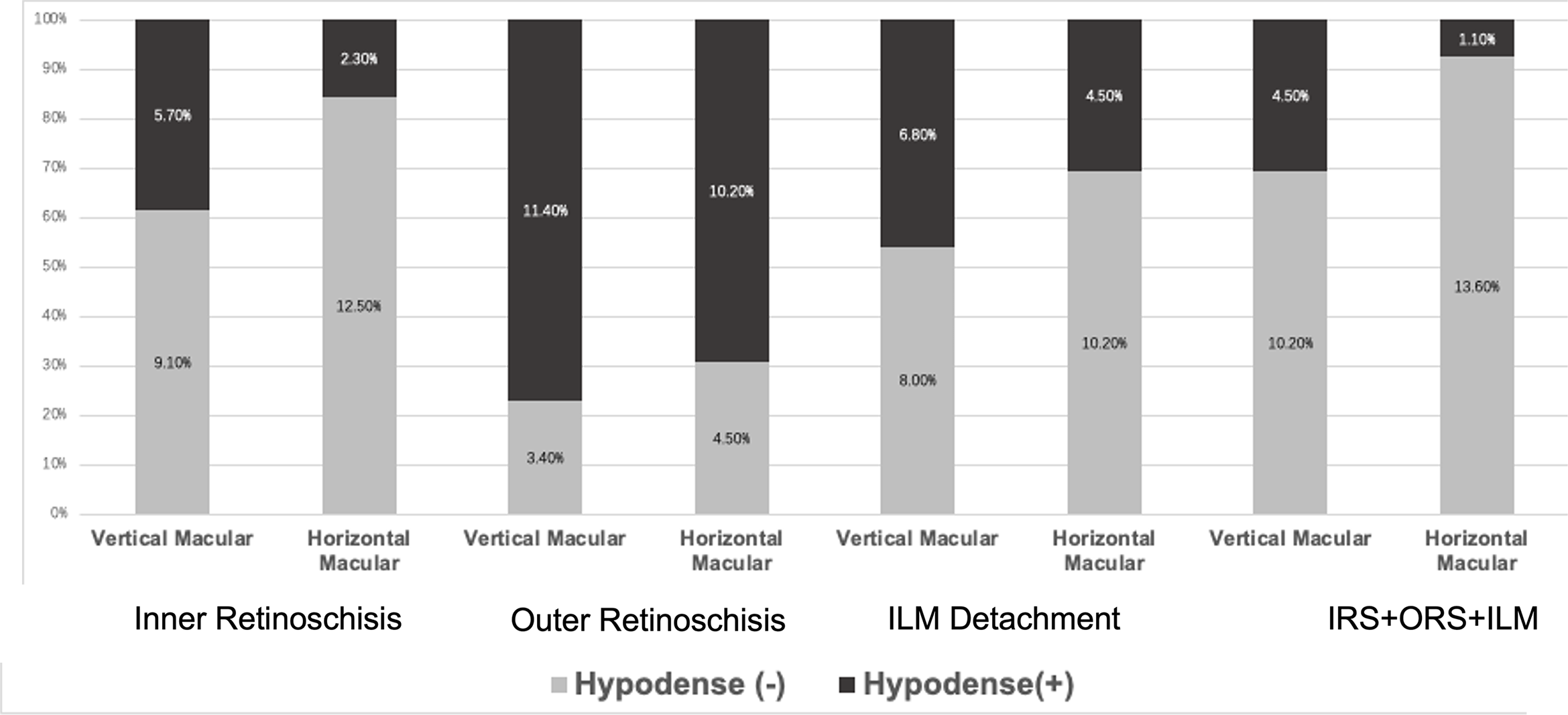

Supplement: Supplementary file 1 [file Image_1.TIF]

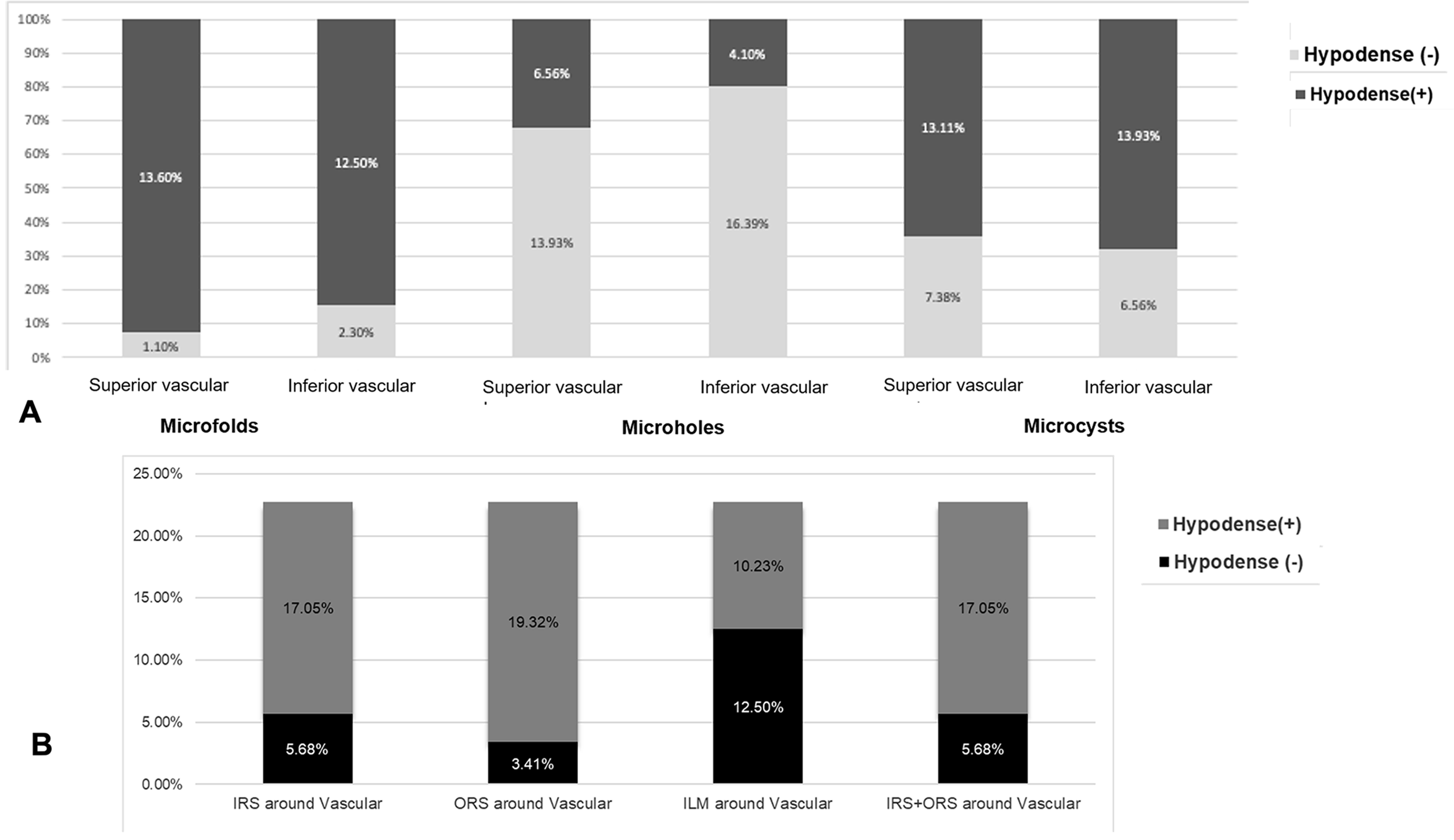

Supplement: Supplementary file 2 [file Image_2.TIF]
